# Prognostic factors for the short-term mortality of patients with rheumatoid arthritis admitted to intensive care units

**DOI:** 10.1186/s41927-020-00164-1

**Published:** 2020-12-04

**Authors:** Toshifumi Fujiwara, Kentaro Tokuda, Kenta Momii, Kyohei Shiomoto, Hidetoshi Tsushima, Yukio Akasaki, Satoshi Ikemura, Jun-ichi Fukushi, Jun Maki, Noriyuki Kaku, Tomohiko Akahoshi, Tomoaki Taguchi, Yasuharu Nakashima

**Affiliations:** 1 Department of Orthopaedic Surgery, Graduate School of Medical Sciences, Kyushu University, 3-1-1, Maidashi, Higashi-ku, Fukuoka-shi, Fukuoka prefecture 812-8582 Japan; 2 Emergency & Critical Care Center, Kyushu University Hospital, Fukuoka-shi, Japan; 3 Intensive Care Unit, Kyushu University Hospital, Fukuoka-shi, Japan

**Keywords:** Rheumatoid arthritis, Prognostic factor, Intensive care unit, Comorbidity, APACHE II, Coagulation abnormality

## Abstract

**Background:**

Patients with rheumatoid arthritis (RA) have high mortality risk and are frequently treated in intensive care units (ICUs).

**Methods:**

This was a retrospective observational study. This study included 67 patients (20 males, 47 females) with RA who were admitted at the ICU of our institution for ≥48 h between January 2008 and December 2017. We analyzed the 30-day mortality of these patients and the investigated prognostic factors in RA patients admitted to our ICU.

**Results:**

Upon admission, the median age was 70 (range, 33–96) years, and RA duration was 10 (range, 0–61) years. The 5-year survival after ICU admission was 47%, and 30-day, 90-day, and 1-year mortality rates were 22, 27, and 37%, respectively. The major reasons for ICU admission were cardiovascular complications (24%) and infection (40%) and the most common ICU treatments were mechanical ventilation (69%), renal replacement (25%), and vasopressor (78%). In the 30-day mortality group, infection led to a fatal outcome in most cases (67%), and nonsurvival was associated with a significantly higher glucocorticoid dose, updated Charlson’s comorbidity index (CCI), and acute physiology and chronic health evaluation (APACHE) II score. Laboratory data obtained at ICU admission showed that lower platelet number and total protein and higher creatinine and prothrombin time international normalized ratio (PT-INR) indicated significantly poorer prognosis. The multivariate Cox proportional hazard model revealed that nonuse of csDMARDs, high updated CCI, increased APACHE II score, and prolonged PT-INR were associated with a higher risk of mortality after ICU admission.

**Conclusion:**

Our study demonstrated that the nonuse of csDMARDs, high updated CCI, elevated APACHE II score, and coagulation abnormalities predicted poorer prognosis in RA patients admitted to the ICU.

## Background

Rheumatoid arthritis (RA) is a systemic autoimmune inflammatory disorder that promotes the production of inflammatory cytokines, which leads to the destruction of joints and systematic complications [1]. Immunosuppressive treatment for RA using glucocorticoids, conventional synthetic disease-modifying antirheumatic drugs (csDMARDs), biologic disease-modifying antirheumatic drugs (bDMARDs), and targeted synthetic disease-modifying antirheumatic drugs (tsDMARDs; e.g., JAK inhibitors) significantly improves disease activity and joint destruction; however, numerous comorbidities and complications, including infection, malignancy, and organ failure (cardiovascular disease [CVD], respiratory distress, and renal failure) remain associated with the increased mortality of RA patients compared with the general population [2–4]. Novel biological treatments have transformed the evolution of joint disease and its complications, such as serious infection [5]. Worsening comorbidities and complications in RA patients require advanced therapy, thus leading to admission into intensive care units (ICUs) [6–9].

A previous study reported that the reasons for the ICU admission of RA patients included CVD and serious infection and showed that RA patients have increased one-year mortality compared with the general population [8]. The prognostic factors for mortality in RA patients admitted to the ICU included admission for infection, higher acute physiology and chronic health evaluation (APACHE) II score [10], and necessity for mechanical ventilation or renal replacement therapy [6–9, 11–14]. Furthermore, the risk factors for the admission of RA patients to an ICU included older age and comorbidities such as chronic obstructive pulmonary disorder (COPD) or chronic kidney disease (CKD) [15]. Considering that patients with systemic rheumatic disease (RD) frequently require ICU treatment for their condition, it is important to understand the prognostic factors [16]. Despite the elevated risk of ICU admission and higher mortality in RA patients, few studies have investigated the prognostic factors for RA patients requiring ICU treatment. Moreover, most studies included multiple autoimmune RDs, such as RA, connective tissue diseases, vasculitis, spondyloarthritis, and other autoimmune disorders, which display different causes of ICU admissions and outcome. A recent study of 43 RA patients admitted to the ICU reported that the risk factors for 30-day mortality included heart failure, liver failure, elevated sequential organ failure assessment (SOFA) score, and vasopressor treatment [17].

RA patients require hospitalization for surgery and various systemic medications owing to joint destruction, flare, or complications and have a high incidence of ICU admission. Thus, this study investigated the prognostic factors for short-term mortality in RA patients after ICU admission.

## Methods

### Patients

This single-center retrospective study reviewed the medical records of all consecutive patients with RA admitted to the ICU of Kyushu University Hospital for ≥48 h between January 1, 2008, and December 31, 2017. Kyushu University Hospital is a 1275-bed national university teaching hospital located at Fukuoka in the south of Japan. This hospital has a critical and emergency care center for patients with severe disorders and/or multiple traumas, as well as an organ transplant center. Our ICU has 10 beds for critical and emergency care and 10 beds for postsurgical and nosocomial severe patients. To identify patients who require intensive care, we excluded patients who were admitted overnight for postoperative observation and analyzed patients who were admitted for ≥48 h. No RA patients died within 48 h after ICU admission in our study. RA diagnosis was established according to the classification criteria of the American College of Rheumatology [18], and patients were examined and verified by a rheumatologist using medical reports or other medical documents in our electronic database. The underlying status of RA at ICU admission included sex, age, RA duration, Steinbrocker stage and class, medication (tsDMARDs, bDMARDs, csDMARDs, and glucocorticoid), and comorbidities to predict mortality by classifying or weighting comorbid conditions according to the updated Charlson’s comorbidity index (CCI) [19, 20]. Steinbrocker stage was graded based on radiographic abnormalities of the hand in patients with RA as follows: stage I, no destructive changes; stage II, osteoporosis and slight cartilage and/or subchondral bone destruction; stage III, osteoporosis and cartilage and/or bone destruction; and stage IV, osseous ankylosis. Steinbrocker class was based on a rating system for physical functional status of patients with RA as follows: class 1, complete physical activity; class 2, adequate activity; class 3, limited activity; and class 4, incapacitated [21, 22]. The updated CCI (0–24 points) was scored based on an index of comorbidities, including congestive heart failure (0–2), dementia (0–2), chronic pulmonary disease (0–1), rheumatic disease (0–1), liver disease (0–4), diabetes with chronic complications (0–1), hemiplegia or paraplegia (0–2), renal disease (0–1), malignancy (0–2), metastatic tumor (0–6), and acquired immune deficiency syndrome (AIDS) / human immunodeficiency virus (HIV) (0–4). Daily glucocorticoid dose at ICU admission was calculated as prednisone dose. The reasons for ICU admission were grouped as cardiovascular complications, infectious complications, liver failure, respiratory disorder, gastrointestinal tract disorder, neurological disorder, renal failure, or other (e.g., trauma and addiction). Complete laboratory data were evaluated at ICU admission and the following day. The APACHE II score (0–71), which predicts the risk of death, was calculated using the patient’s age, previous health status, and routine physiologic measurements, including body temperature, mean arterial blood pressure, heart rate, respiratory rate, arterial pH, alveolar–arterial oxygen difference, or partial pressure of oxygen in arterial blood, depending on the fraction of the inspired oxygen, serum sodium and potassium, creatinine, hematocrit, white blood cell count, and Glasgow Coma Scale score during the first 24 h following ICU admission [23]. Disseminated intravascular coagulation (DIC) score [24, 25] at ICU admission was made by the Japanese Association for Acute Medicine DIC scoring system using prothrombin time international normalized ratio (PT-INR) and fibrin degradation product [26]. Several intensive treatments were performed for organ failure such as mechanical ventilation, renal replacement therapy, plasma exchange therapy, vasopressors, and/or antibiotic therapy. The duration of each ICU and hospital stay was documented. This retrospective study was approved by the Ethics Committee of Kyushu University Hospital (approval number: 30–478).

### Outcome measures

The primary outcomes were the 30-day, 90-day, and 1-year survival rates of all patients included in the study. Information on patient survival was obtained from medical records at our hospital and/or changing hospital after 30 days, 90 days, and 1 year. A total of 67 patients were followed up at 30 and 90 days, but 5 patients were lost to follow-up 1 year after ICU admission because the patients changed hospital or did not return to the hospital.

### Statistical analysis

Statistical analysis was performed using JMP pro 13.0.0 (SAS Institute, Cary, NC). Categorical variables in 30-day mortality were compared using Pearson’s chi-squared test, and continuous variables were analyzed by the Mann–Whitney *U* test. The survival of patients with RA after ICU admission was analyzed according to the Kaplan–Meier method with computation of 95% confidence intervals (CIs). To identify the independent predictors of mortality in patients with RA admitted to ICU, the Cox proportional hazards model was used. The variables for multivariable analysis were selected from the statistically significant variables identified by univariable analysis using the Lasso approach. Statistical difference was defined as *P* < 0.05 for all comparisons. All data were presented as mean ± standard deviation (median, range).

## Result

### Baseline characteristics of patients

**Fig. 1:**
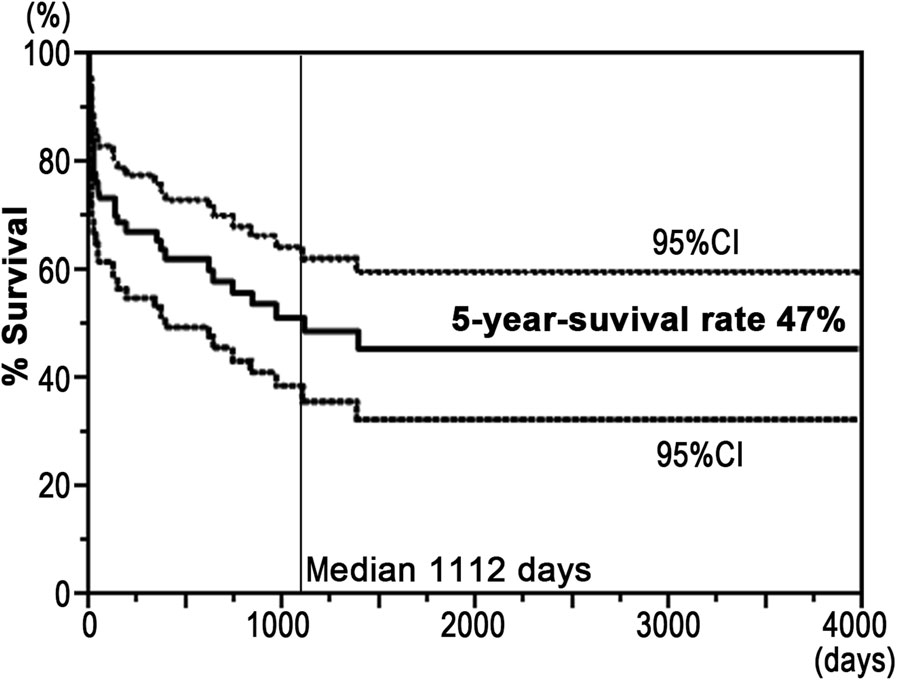
Overall survival of 67 patients after first admission to our ICU. The 5-year survival rate was 47%, and the median survival time was 1112 days (range, 3–4380 days). Overall survival is represented by a solid line, and 95% CIs are represented by broken lines

This study included 67 consecutive patients with RA (*n* = 67) who were admitted to the ICU at Kyushu University Hospital between January 1, 2008, and December 31, 2017 (Table 1). Six of the patients were readmitted to the ICU more than once during this period but were only analyzed at the first ICU admission. The mean age at ICU admission was 68 ± 13 years old (range, 33–96, median, 70), the median RA duration was 14 ± 15 years (range, 0–61, median, 10), and the average follow-up duration was 954 ± 1073 days (range, 3–4380, median, 646). Kaplan–Meier survival curve analysis revealed that 5-year survival after the first ICU admission was 47% (median, 1112 days) (Fig. 1), and the 30-day, 90-day, and 1-year mortality rates were 22% (15/67), 27% (18/67), and 37% (23/62), respectively (Table 1). Given that the majority of nonsurvivors died within the first 30 days, we investigated the prognostic factors of 30-day mortality by using univariate analysis. Table 1 shows the characteristics associated with RA status at baseline. There was no statistical difference between the 30-day mortality at baseline of survivors and nonsurvivors. Treatment with bDMARDs (*P* = 0.1680) or csDMARDs (*P* = 0.4493) immediately prior to ICU admission showed no statistical difference, whereas the use of glucocorticoids (*P* = 0.0239) was associated with poorer prognosis in nonsurvivors in the 30-day mortality group in a glucocorticoid (conversion of prednisone) dose-dependent manner (*P* = 0.0095). After ICU admission, patients who were able take csDMARDs and/or glucocorticoid continued with this medication, whereas those who were unable to take anything by mouth were administered a corresponding amount of glucocorticoid without csDMARDs via injection until they could take oral medicine. Treatment with bDMARDs was temporally discontinued during the ICU stay. Most patients, who had been recovered from critical condition and could take orally, has begun taking same amount of DMARDs again. bDMARD contained infliximab, etanercept, and tocilizumab, and csDMARDs were methotrexate, tacrolimus, salazosulfapyridine, bucillamine, mizoribine, iguratimod, and cyclosporine. No patient had been treated with tsDMARDs.

**Table 1:** Rheumatoid arthritis characteristics of the 30-day mortality group at ICU admission

Characteristic	All patients (*n* = 67)	30-day mortality		
		Survivors (*n* = 52)	Nonsurvivors (*n* = 15)	*P*-value
Age (years)	68.3 ± 13.5 (70, 33–96)	66.9 ± 14.4 (70, 33–96)	73.1 ± 8.5 (75, 54–85)	0.1484
Sex (female/male)	47/20 (70%/30%)	39/13 (75%/25%)	8/7 (53%/47%)	0.1062
RA duration (years)	13.9 ± 15.0 (10, 0–61)	14.1 ± 15.7 (9, 0–61)	12.9 ± 12.9 (10, 1–48)	0.7120
Steinbrocker				
Stage (I/II/III/IV)	9/19/18/21 (13%/28%/27%/31%)	9/15/14/17 (13%/29%/27%/31%)	2/4/4/5 (13%/27%/27%/33%)	0.9975
Class (1/2/3/4)	7/16/38/6 (10%/24%/57%/9%)	8/14/29/4 (12%/25%/56%/8%)	1/3/9/2 (7%/20%/60%/13%)	0.8407
Medication				
bDMARDs	6 (9%)	6 (12%)	0 (0%)	0.1680
csDMARDs	37 (55%)	30 (57.7%)	7 (46.7%)	0.4493
MTX	19 (28%)	17 (33%)	2 (13%)	0.1428
MTX dose (mg)	6.4 ± 2.1 (6, 4–12)	6.2 ± 1.7 (6, 4–8)	8.0 ± 5.7 (8, 4–12)	0.7267
Others^a^	24 (36%)	18 (35%)	6 (40%)	0.7016
Glucocorticoids	53 (79%)	38 (73.1%)	15 (100%)	**0.0239**
Glucocorticoid dose (mg) (conversion of prednisone)	5.3 ± 5.3 (5, 0–25)	4.5 ± 5.0 (4, 0–25)	8.1 ± 5.8 (7, 1–25)	**0.0095**
30-day mortality	15/67 (22%)			
90-day mortality	18/67 (27%)			
1-year mortality	23/62 (37%)			

Data represent mean ± SD (median, range)

*bDMARDs* biologic disease-modifying anti-rheumatic drugs, *csDMARDs* conventional synthetic disease-modifying anti-rheumatic drugs, *MTX* methotrexate, *RA* rheumatoid arthritis

^a^Others of csDMARDs contained tacrolimus, salazosulfapyridine, bucillamine, mizoribine, iguratimod, and cyclosporine

### Comorbidities with RA patients at ICU admission

The comorbidities of the RA patients were calculated using updated CCI, which predicts hospital mortality by classifying or weighting comorbidities [19, 20] (Table 2). The total CCI score (*P* = 0.0001), including the liver disease (*P* = 0.0004) and renal disease scores (*P* = 0.0009), was significantly increased in nonsurvivors compared with survivors.

**Table 2:** The comorbidities and status of patients with RA at ICU admission

	All patients (*n* = 67)	30-day mortality		
		Survivors (*n* = 52)	Nonsurvivors (*n* = 15)	*P* value
Updated CCI				
Congestive heart failure (0/2)	0.3 ± 0.7 (0, 0–2)	0.2 ± 0.6 (0, 0–2)	0.5 ± 0.9 (0, 0–2)	0.0904
Dementia (0/2)	0.09 ± 0.4 (0, 0–2)	0.04 ± 0.3 (0, 0–2)	0.3 ± 0.7 (0, 0–2)	0.0617
Chronic pulmonary disease (0/1)	0.3 ± 0.5 (0, 0–2)	0.3 ± 0.5 (0, 0–2)	0.4 ± 0.5 (0, 0–2)	0.5056
Rheumatologic disease (0/1)	1	1	1	ns
Liver disease (0/2/4)	0.6 ± 1.3 (0, 0–4)	0.2 ± 0.8 (0, 0–4)	1.7 ± 2.0 (0, 0–4)	0.0004
Diabetes with chronic complications (0/1)	0.2 ± 0.6 (0, 0–1)	0.2 ± 0.6 (0, 0–1)	0.1 ± 0.5 (0, 0–1)	0.5896
Hemiplegia/paraplegia (0/2)	0.2 ± 0.6 (0, 0–2)	0.2 ± 0.6 (0, 0–2)	0 (0, 0)	0.1712
Renal disease (0/1)	0.3 ± 0.5 (0, 0–1)	0.2 ± 0.4 (0, 0–1)	0.7 ± 0.5 (1, 0–1)	0.0009
Malignancy (0/2/6)	0.3 ± 0.9 (0, 0–6)	0.3 ± 0.7 (0, 0–2)	0.4 ± 1.5 (0, 0–6)	0.5383
AIDS/HIV (0/4)	0	0	0	ns
Total CCI (0–24)	3.3 ± 2.0 (3, 1–12)	2.7 ± 1.4 (3, 1–6)	5.1 ± 2.5 (4, 2–12)	0.0001
APACHE II (0–71)	16.2 ± 7.1 (15, 2–35)	14.6 ± 5.6 (14, 2–25)	21.7 ± 9.2 (24, 2–35)	0.0029
ICU stay (days)	7.3 ± 7.7 (4, 2–39)	6.6 ± 7.5 (4, 2–39)	9.4 ± 8.4 (5, 2–30)	0.1153
Hospitalization (days)	42.1 ± 35.1 (30, 3–176)	46.2 ± 36.5 (34, 5–176)	27.8 ± 25.6 (24, 3–104)	0.0302
DIC score	3.6 ± 2.6 (3, 1–11)	2.8 ± 1.9 (2, 1–11)	6.4 ± 2.9 (7, 2–11)	< 0.0001
Reason for ICU admission				0.3239
Cardiovascular disease	16 (24%)	15 (29%)	1 (7%)	
Infection	27 (40%)	17 (33%)	10 (67%)	
Liver failure	2 (3%)	1 (2%)	1 (7%)	
Respiratory disorder	9 (13%)	7 (13%)	2 (13%)	
Gastrointestinal tract disorder	1 (1%)	1 (2%)	0 (0%)	
Neurological disorder	4 (6%)	4 (8%)	0 (0%)	
Renal failure	3 (4%)	3 (6%)	0 (0%)	
Others	5 (7%)	4 (8%)	1 (7%)	
ICU treatment				
Mechanical ventilation	46 (69%)	34 (65%)	12 (80%)	0.2824
Renal replacement therapy	17 (25%)	8 (15%)	9 (60%)	0.0005
Plasma exchange therapy	3 (4%)	1 (2%)	2 (13%)	0.0598
Vasopressor therapy	52 (78%)	40 (77%)	12 (80%)	0.8012
Antibiotic therapy	39 (58%)	27 (52%)	12 (80%)	0.0521

Data represent means ± SD (median, range)

*AIDS* acquired immunodeficiency syndrome, *APACHE* II acute physiology and chronic health evaluation II, *CCI* Charlson’s comorbidity index, *DIC* disseminated intravascular coagulation, *HIV* human immunodeficiency virus, *ICU* intensive care unit, *ns* not significant

### Reasons for ICU admission and treatments

The analysis of ICU scores revealed that high APACHE II scores were significantly associated with 30-day mortality (*P* = 0.0029). The duration of ICU stay showed no difference between nonsurvivors and survivors groups, whereas hospitalization was statistically shorter in the 30-day mortality group owing to death within 30 days. The main reason for ICU admission was infection (40%; 27/67) (sepsis, 8; respiratory, 8; gastrointestinal, 4; Hepatic 3; Cardiovascular 2; meningitis 1; Muscle 1) (Table 3), followed by cardiovascular complications (24%; 16/67) (acute myocardial infarction, 3; acute aortic dissection, 2; post heart operative management, 11), respiratory disorder (13%; 9/67) (acute adult respiratory distress syndrome, 3; others, 6), neurological disorder (6%; 4/67) (cerebral infarction, 2; intracerebral hemorrhage, 1; transient cerebral ischemia, 1), renal failure (4%; 3/67) (post renal transplant, 3), liver failure (3%; 2/67) (post hepatic transplant, 2), gastrointestinal tract disorder (1%; 1/67) (post esophageal cancer operative management, 1), and others (7%; 5/67). Among these reasons, infection was the leading cause of death in the 30-day mortality group (67%; 10/15), and the causes of infection in the 30-day mortality group were sepsis (40%; 4/10), bacterial pneumonia (30%; 3/10), and viral hepatitis (30%; 3/10) (Table 3). The reason of ICU treatments included mechanical ventilation (69%; 47/67), renal replacement therapy (25%; 17/67), plasma exchange therapy (3/67; 4%), vasopressor, therapy (52/67, 78%), and antibiotic therapy (39/67; 58%) in the survivor and nonsurvivor groups. Renal replacement therapy for renal failure associated with infection or other organ failures was statistically elevated in nonsurvivors compared with survivors (*P* = 0.0005) (Table 2).

**Table 3:** Sites of serious infections

		Sepsis	Respiratory		Gastrointestinal	Hepatic	Cardiovascular	Meningitis	Muscle	Total
		Bacteremia	Bacterial	Fungal	Bacterial	Viral	Bacterial	Bacterial	Bacterial	
Total		8 (30%)	6 (22%)	2 (7%)	4 (15%)	3 (11%)	2 (7%)	1 (4%)	1 (4%)	27 (100%)
30-day mortality	Survivors	4 (50%)	3 (50%)	2 (100%)	4 (100%)	0 (0%)	2 (100%)	1 (100%)	1 (100%)	17 (63%)
	Nonsurvivors	4 (50%)	3 (50%)	0 (0%)	0 (0%)	3 (100%)	0 (0%)	0 (0%)	0 (0%)	10 (37%)

### Biomarkers at ICU admission

To identify the prognostic biomarkers at ICU admission, we performed univariate analysis to compare the difference between survivors and nonsurvivors in the 30-day mortality groups by using laboratory tests at the first and second days after ICU admission. Mann–Whitney analysis uncovered that the nonsurvivors in the 30-day mortality group showed significantly lower platelet number, total protein, and albumin and higher blood urea nitrogen, creatinine, and PT-INR. In particular, lower platelet number and PT-INR were significant on both the first and second days (Table 4). Furthermore, DIC score was higher in the nonsurvivors than in the survivors according to the coagulation abnormalities (Table 2).

**Table 4:** Laboratory data at ICU admission

	30-day mortality					
	ICU admission day 1			ICU admission day 2		
	Survivors (*n* = 52)	Nonsurvivors (*n* = 15)	*P*-value	Survivors (*n* = 52)	Nonsurvivors (*n* = 15)	*P* value
White blood cell (/μL)	10,346 ± 5830 (8990, 400–25,630)	13,549 ± 7284 (12,000, 4510–33,900)	0.0810	11,433 ± 5106 (10,845, 740–27,740)	13,577 ± 8145 (12,150, 6470–39,990)	0.4890
Hemoglobin (g/dL)	10.5 ± 2.2 (10.9, 5.8–13.8)	10.5 ± 2.2 (10.1, 7.7–16.0)	0.6572	10.2 ± 1.7 (10.5, 6.8–14.0)	10.3 ± 2.3 (10.2, 5.6–13.9)	0.9281
Platelet (× 10^3^/μL)	178 ± 120 (137, 25–567)	113 ± 93 (111, 20–371)	0.0365	159 ± 110 (126, 16–586)	98 ± 63 (97, 11–211)	0.0446
C-reactive protein (mg/dL)	6.3 ± 7.6 (3.9, 0.01–28.7)	11.6 ± 15.0 (7.1, 0.1–51.0)	0.1552	10.1 ± 8.0 (9.3, 0.06–27.0)	11.6 ± 15.2 (7.3, 0.2–58.3)	0.6410
Total protein (g/dL)	6.0 ± 1.1 (6.3, 3.7–8.0)	5.0 ± 1.3 (4.8, 2.0–7.3)	0.0063	5.5 ± 0.9 (5.6, 3.3–7.1)	5.0 ± 0.8 (4.8, 4.0–6.4)	0.0540
Albumin (g/dL)	3.2 ± 0.8 (3.3, 1.4–4.6)	2.4 ± 0.8 (2.3, 0.7–3.8)	0.0016	3.0 ± 0.7 (2.9, 1.5–5.0)	2.7 ± 0.6 (2.8, 1.8–3.7)	0.3947
Blood urea nitrogen (mg/dL)	27.9 ± 18.0 (22, 4–95)	44.6 ± 34.7 (35, 2–118)	0.0758	28.8 ± 17.8 (24, 10–77)	46.5 ± 30.3 (37, 6–116)	0.0139
Creatinine (mg/dL)	1.5 ± 1.5 (0.9, 0.4–7.6)	2.5 ± 2.4 (1.9, 0.6–10.0)	0.0665	1.4 ± 1.2 (0.9, 0.3–5.1)	2.3 ± 1.6 (1.8, 0.7–6.3)	0.0101
Total bilirubin (mg/dL)	0.9 ± 0.7 (0.8, 0.1–4.1)	3.6 ± 4.5 (0.9, 0.2–12.7)	0.4973	1.1 ± 0.9 (0.9, 0.2–4.8)	3.7 ± 4.4 (1.4, 0.2–12.1)	0.1398
Lactate dehydrogenase (U/L)	367.3 ± 249.6 (314, 112–1439)	506.3 ± 413.7 (409, 206–1953)	0.1011	348.8 ± 166.4 (312, 100–837)	604.0 ± 583.9 (424, 189–2159)	0.1027
PT-INR	1.2 ± 0.2 (1.1, 0.9–2.2)	1.7 ± 1.0 (1.5, 1.1–5.0)	0.0003	1.2 ± 0.5 (1.1, 0.9–4.2)	1.4 ± 0.4 (1.2, 1.0–2.7)	0.0171

Data represent means ± SD (median, range)

*ICU* intensive care unit, *PT-INR* prothrombin time-international normalized ratio

### Prognostic factors for mortality in RA patients transferred to the ICU

Table 5 shows the univariate and multivariate Cox proportional hazards models for mortality after ICU admission. The significant factors after univariate analyses, including glucocorticoid dose, updated CCI, APACHE II score, platelet number, and PT-INR, and previously reported prognostic factors, including sex, age, duration of RA, and use of csDMARDs or b/tsDMARDs, [6, 8, 15] were selected using the Lasso approach. This was done after the analysis of the prognostic factors for survival after ICU admission from the isolated variables (use of csDMARDs, glucocorticoid dose, updated CCI, APACHE II score, platelet number, and PT-INR) using the multivariate Cox proportional hazards model. The use of csDMARDs (hazard ratio [HR], 0.413; 95% CI, 0.190–0.899; *P* = 0.0229), elevated updated CCI (HR, 1.522; 95% CI, 1.203–1.892; *P* = 0.0007), high APACHE II score (HR, 1.045; 95% CI, 1.045–1.184; *P* = 0.0008), and extended PT-INR (HR, 2.670; 95% CI 1.411–4.737; *P* = 0.0051) were associated with an increased risk of mortality after ICU admission.

**Table 5:** Univariate and Multivariate analyses of overall survival after ICU admission in patients with RA

	Univariate Analysis			Multivariate Analysis		
	HR	95% CI	*P*-value	HR	95% CI	*P*-value
csDMARDs (yes)	0.569	0.283–1.146	0.1147	**0.413**	**0.190–0.899**	**0.0229**
Prednisone (higher dose)	**1.072**	**1.015–1.022**	**0.0148**	1.052	0.989–1.112	0.1062
Total CCI	**1.511**	**1.265–1.795**	**< 0.0001**	**1.522**	**1.203–1.892**	**0.0007**
APACHE II	**1.115**	**1.053–1.181**	**0.0002**	**1.112**	**1.045–1.184**	**0.0008**
Platelet number	1.011	0.981–1.039	0.4495	1.020	0.989–1.047	0.2023
PT-INR	**2.020**	**1.238–2.911**	**0.0007**	**2.670**	**1.411–4.737**	**0.0051**

*95% CI* 95% confidence interval, *HR* Hazard ratio, *CCI* Charlson’s comorbidity index, *PT-INR* prothrombin time-international normalized ratio

## Discussion

This study retrospectively analyzed the prognosis of 67 RA patients after ICU admission and identified the predictive factors of mortality. We elucidated that the prognostic factors of mortality for RA patients admitted to ICU included the use of csDMARDs, elevated updated CCI, high APACHE II score, and prolonged PT-INR at ICU admission.

Previous studies have examined the risk factors and mortality of RA patients after ICU treatment [6–9, 11–15, 17, 27, 28]. The short-term fatal outcome of RD, including RA, tended to be worse in RA patients than in the general population [9, 17, 27], and the long-term (1–3 years) mortality of RA patients was significantly increased after ICU admission [8, 27]. In our institution, the 30-day, 90-day, and 1-year mortalities after ICU admission were 21, 27, and 37%, each. This is likely due to the tertiary nature of our hospital and because our study design excluded mild cases. Furthermore, Peschken et al. reported that the most common reasons for ICU admission were CVD and infection [8]. CVD in patients with RA is known to be associated with a higher prevalence of comorbidity and is a cause of mortality compared with that in the general population [29, 30], whereas CVD mortality has been declining due to the current strict disease control measures [31]. The percentage of patients admitted to the ICU for CVD was lower than that for serious infections because of the possibility of a favorable response to the treatment and exclusion of patients staying for less than 24 h. A French cohort study involving patients with RD demonstrated that infection and RD exacerbation were the most common causes for ICU admission [13]. Barrett et al. reported higher rates of severe sepsis and poorer prognosis in RA patients than in the general population [27]. Patients with RA have an increased a risk of infection [2–4, 15, 17], and the most frequent site of infection is the respiratory system, followed by soft tissue, bone, and joint [5, 32]. In our study, due to the critical condition of the patients that required treatment in the ICU, sepsis (8, 30%) and respiratory infection (8, 30%) were the most common infections, as shown in Table 3. Viral hepatitis was observed in three nonsurvivors, all of whom had hepatitis B virus (HBV) reactivation. Although the reported HBV-related mortality ranges from 1.9 to 4.0% in Taiwanese patients with RA [33], all three patients with HBV reactivation in our study died of liver failure, suggesting that the prevention of HBV reactivation is important. A previous study proved that the risk factors for the ICU admission of RA patients due to infection included the non-use of csDMARDs, old age, and comorbidity with COPD or CKD [15]. The current study found that the majority of the 30-day mortality group admitted to our ICU due to infection was nonsurvivors. Immunosuppressive treatments, including bDMARDs, csDMARDs, and glucocorticoids, are reportedly associated with infection [15, 34–39], whereas we found that the use of csDMARDs reduced the risk of mortality after ICU admission using the multivariate Cox proportional hazards model. Additionally, despite the lack of a significant difference in dose-related risk of glucocorticoids in the multivariate analysis, a higher glucocorticoid dose appeared to be associated with an increased risk of mortality. Based on previous reports on the risks related to infections and medications, including glucocorticoid, csDMARDs, and bDMARDs [6, 15, 17, 35, 36, 39–41], a dose-related glucocorticoid was reported to increase the risk of infection, whereas csDMARDs were reported to reduce the risk and mortality in RA [15, 42, 43]. Treatment with glucocorticoids raised the incidence and hazard of adverse effects in RA patients, such as diabetes mellitus, osteoporosis, thrombotic stroke, CVD, serious infection, and death [44–46]. Two studies previous showed that the use of glucocorticoids for RD led to poorer prognosis in short-term outcome after ICU admission [28, 47]. Conversely, increased glucocorticoid doses might have been used in patients with higher disease activity who could not be treated with csDMARDs and/or b/tsDMARDs; however, we were unable to accurately analyze the disease activity in the patients because of altered consciousness or the ICU settings. Furthermore, a short course of glucocorticoids with methotrexate could lead to a low disease activity earlier [48]. Since several studies have reported high disease activity and early presence of joint damage as poor prognostic factors for RA [49–51], treatment with csDMARDs is necessary for prompt RA control. Together with previous reports, our results suggest that RA disease activity might be controllable using csDMARDs and minimum glucocorticoid doses, which might reduce mortality after ICU admission. In our study, most of the patients with RA (79%) had been treated with glucocorticoid due to various severe complication, such as heart, liver, and renal failure, suggesting that the patients who had been hard to use csDMARDs and decrease the amount of glucocorticoid for their disease activity and severe complication might be poorer prognosis. Actually, only 55% of our cohort had been treated with csDMARDs, suggesting that the patients, who had difficulty to use csDMRDs because of various reasons including some adverse event and comorbidities, were possible to be required the treatment at ICU.

RA patients often have several comorbidities, and previous studies have reported the risk factors for ICU admission and mortality, such as pulmonary disorder, renal dysfunction, and hypertension [6, 13, 15]. To evaluate comorbidities, we calculated the updated CCI, which can be used to predict hospital mortality [20]. Univariate analysis showed that the updated CCI, particularly the liver and renal failure scores, was higher for nonsurvivors in the 30-day mortality group than for survivors (Table 2) [6, 13, 15, 17]. In addition, a higher updated CCI was found to be associated with an elevated risk of mortality after ICU admission using the multivariate Cox proportional hazards model. Given that nearly 80% of patients with RA suffer from comorbidities [52, 53], a higher updated CCI, which was developed for the prediction of hospital mortality [19, 20], might be a helpful predictor of poor prognosis in patients with RA after ICU admission. In the current study, the majority of RA patients were treated with mechanical ventilation and vasopressor therapy after ICU admission. Similar to another study [12], there was a significantly greater use of renal replacement therapy in the nonsurvivors of the 30-day mortality group than in the survivors. Renal replacement therapy was frequently used for worsened liver failure and renal disease among comorbidities and was increased in the nonsurvivors of the 30-day mortality group. Together with the findings from previous reports [12, 54], our data indicate that ICU patients requiring renal replacement therapy showed poorer prognosis. Previous studies reported that other organ replacement therapies, including mechanical ventilation and vasopressors, were associated with a higher mortality in the ICU population [12, 17, 47, 55, 56]. By contrast, the present study did not find any difference in the 30-day mortality groups probably because our study only included RA patients and did not include those with other collagen diseases, such as systemic lupus erythematosus, dermatomyositis, Sjögren’s disease, progressive systemic sclerosis, mixed connective tissue disease, or vasculitis.

Some studies have reported that the prognostic factors of ICU mortality in patients with RD included high APACHE II and SOFA scores, serious infection, mechanical ventilation, vasopressor, renal replacement therapy, and glucocorticoid dose [6, 7, 9, 11, 12, 17, 28]. The APACHE II and SOFA scores, which were predictive factors of ICU mortality in the general population, have also predicted ICU mortality in RA patients [6, 7, 11, 12, 14, 23]. In line with previous studies [11, 57–59], our multivariate analysis also identified an elevated APACHE II score as a predictor of mortality. In fact, in our study including only patients with RA, the 30-day mortality of the nonsurvivors was significantly higher than that of the survivors (21.7 ± 9.2 vs 14.6 ± 5.6). So far, only one report [17] has investigated the APACHE II score specifically in patients with RA, and our study demonstrated that the association between APACHE II score and mortality of RA is similar to that in previous reports on RD. Considering that the APACHE II scores of RD patients were shown to be approximately equal to those of the general population due to their critical condition needed intensive care [14, 17], the results of the current study show that the APACHE II score in RA patients was also a prognostic factor of ICU mortality. Finally, laboratory tests showed that coagulation abnormalities were a prognostic biomarker associated with poor outcome in all ICU mortality [60]. The multivariate Cox proportional hazards model demonstrated that prolonged PT-INR at ICU admission could predict ICU mortality using a routine coagulation test. Therefore, PT-INR should be extended to the ICU admission of patients treated with an anticoagulation agent for their comorbidity. The DIC scores and PT-INR on the day after ICU admission were significantly elevated in the nonsurvivors of the 30-day mortality group, thus suggesting that PT-INR, as a representative of DIC, is a useful biomarker for ICU survival. Indeed, several studies have reported that the DIC score correlated with several scoring systems used in the ICU, such as APACHE II score [61, 62]. On the other hand, Awgstwurm *et*. *al* demonstrated that the DIC and APACHE II scores were independently correlated with survival time [63]. Additionally, their study revealed that the combination of the APACHE II score with the coagulation abnormality score was a better predictor of mortality than the APACHE II score alone. In our study, PT-INR had no correlation with APACHE II score (*R* = − 0.1363, *P* = 0.2714), suggesting that prolonged PT-INR in combination with the APACHE II score might predict mortality in patients with RA admitted to the ICU.

This study has several limitations. First, clinical data were retrospectively analyzed. Second, this study used a small cohort from a single institution and did not include a control group. However, the characteristics of the patients treated at the ICU vary between different institutions. Our study was able to analyze an RA population with more critical comorbidities and complications because our institution treated patients with more severe disorders, such as organ transplantation, compared with other institutions around this area. Third, we did not analyze the disease activity of all RA patients, and this approach may have affected the interventions and outcomes of the patients. However, it was difficult to identify disease activity because our study included patients with impaired consciousness and/or are immobile.

## Conclusion

In summary, our study showed that RA patients admitted to the ICU have high 30-day (21%), 90-day (27%), and 1-year (37%) mortality. Comorbidities, such as liver disease and renal failure, increased the risk of mortality, and most patients requiring renal replacement therapy had a fatal outcome. Infectious complications were the highest in the nonsurvivors of the 30-day mortality group. The multivariate analysis revealed that the risk factors for mortality in patients with RA admitted to the ICU are nonuse of csDMARDs, high updated CCI, elevated APACHE II score, and prolonged PT-INR.

## Data Availability

The datasets generated during and/or analyzed during the current study are available from the corresponding author on reasonable request.

## Abbreviations

RA: Rheumatoid arthritis
RD: Rheumatic disease
csDMARDs: Conventional synthetic disease-modifying antirheumatic drugs
bDMARDs: Biologic disease-modifying antirheumatic drugs
tsDMARDs: Targeted synthetic disease-modifying antirheumatic drugs
CVD: Cardiovascular disease
COPD: Chronic obstructive pulmonary disorder
CKD: Chronic kidney disease
HBV: Hepatitis B virus
ICU: Intensive care unit
APACHEII: Acute physiology and chronic health evaluation II
SOFA: Sequential organ failure assessment
CCI: Charlson’s comorbidity index
DIC: Disseminated intravascular coagulation
PT-INR: Prothrombin time international normalized ratio
